# Lacrimal gland atrophy and dry eye related to isotretinoin, androgen, and prolactin: differential diagnosis for Sjögren’s syndrome

**DOI:** 10.5935/0004-2749.20210012

**Published:** 2025-02-02

**Authors:** Amanda Pires Barbosa, Fabíola Reis de Oliveira, Flavio Jaime da Rocha, Valdair Francisco Muglia, Eduardo Melani Rocha

**Affiliations:** 1 Department of Ophthalmology, Otorhinolaryngology and Head & Neck Surgery, Faculdade de Medicina de Ribeirão Preto, Universidade de São Paulo, Ribeirão Preto, SP, Brazil; 2 Department of Medicine, Faculdade de Medicina de Ribeirão Preto, Universidade de São Paulo, Ribeirão Preto, SP, Brazil; 3 Department of Ophthalmology, Faculdade de Medicina de Uberlândia, Universidade Federal de Uberlândia, Uberlândia, MG, Brazil

**Keywords:** Testosterone congeners, Isotretinoin, Dry eye syndrome, Lacrimal glands, Magnetic resonance imaging, Pituitary neoplasms, Adenoma, Prolactin, Sjögren’s syndrome, Congêneres da testosterona, Isotretinoína, Síndromes do olho seco, Glândulas lacrimais, Imagem por ressonância magnética, Neoplasias hipofisárias, Adenoma, Prolactina, Síndrome de Sjögren

## Abstract

This report is of three cases of sicca syndrome, initially suspected to be Sjögren’s syndrome, which was ruled out by clinical and laboratory investigations. The patients were a 24-year-old woman, a 32-year-old man, and a 77-year-old woman with chronic symptoms of sicca syndrome, including dry eye syndrome. The first case was associated with the use of isotretinoin, a retinoic acid. The second was associated with the use of anabolic androgenic steroids, and the third was related to a prolactinsecreting pituitary adenoma. All cases manifested sicca, including dry eye syndrome, after those events, and the manifestations persisted. Magnetic resonance imaging revealed bilateral atrophy of the lacrimal gland. The medical history, ocular examinations, laboratory exams, and magnetic resonance images confirmed dry eye syndrome; however, the exams were all negative for Sjögren’s syndrome. The lacrimal gland was absent on magnetic resonance imaging in all three cases. The clinical history revealed that the signs and symptoms appeared after chronic exposure to retinoic acid, anabolic androgenic steroids, and a prolactin-secreting pituitary adenoma, respectively. Chronic isotretinoin, anabolic androgenic steroids, and prolactin-secreting pituitary adenoma or, in this last case, its inhibitory treatment, can cause lacrimal gland atrophy, sicca syndrome, and dry eye syndrome, and a differential diagnosis of Sjögren’s syndrome. Further studies on doses, time, and other susceptibilities to the long-lasting adverse effects of retinoic acid, anabolic androgenic steroids, and the repercussions of prolactin-secreting pituitary adenoma are necessary to confirm and expand upon these associations.

## Abstract (pt)

O relato descreve três casos de síndrome de sicca, inicialmente suspeitos de serem a síndrome de Sjögren, que foram negados pela investigação clínica e laboratorial. O primeiro associado ao uso de isotretinoína, um ácido retinóico, o segundo ao uso de esteroides androgênicos anabolizantes e o terceiro relacionado ao adenoma da hipófise secretora da prolactina, todos manifestaram sicca, incluindo a síndrome do olho seco após esses eventos e as manifestações persistem. A ressonância magnética revelou atrofia bilateral da glândula lacrimal. Eles eram uma mulher de 24 anos, um homem de 32 anos e uma mulher de 77 anos com sintomas crônicos da síndrome de sicca, incluindo a síndrome do olho seco. A história médica, o exame ocular, os exames laboratoriais e a ressonância magnética foram confirmados como síndrome do olho seco, no entanto, todos os exames foram negativos para a síndrome de Sjögren. A glândula lacrimal estava ausente na ressonância magnética nos três casos. A história clínica revelou que sinais e sintomas se manifestaram após exposição crônica ao ácido retinóico, esteróides anabolizantes androgênicos e adenoma secretivo da prolactina hipofisária, respectivamente. Isotretinoína crônica, esteroides anabólicos androgênicos e adenoma hipofisário secretor de prolactina ou, neste último caso, seu tratamento inibitório pode ser a causa da atrofia da glândula lacrimal, síndrome da sicca e síndrome do olho seco e diagnóstico diferencial da síndrome de Sjögren. Estudos adicionais sobre doses, duração e outras suscetibilidades aos efeitos adversos duradouros do ácido retinóico, esteroides androgênicos anabólicos e repercussões do adenoma da hipófise secretora da prolactina são necessários para confirmar e detalhar essas associações.

## INTRODUCTION

Retinoic acid (RA), anabolic androgen steroids (AAS), and prolactin (PRL) act on the main lacrimal gland (LG), meibomian glands (MG), and the ocular surface (OS) epithelia. Therefore, that they have physiological effects on these tissues’ homeostasis and a potential therapeutic effect on dry eye syndrome (DES)^(1-4)^. Conversely, genetic predisposition, hormone interactions, and excessive exposure to those hormones can induce paradoxical effects in the OS or other exocrine tissues, as previously reported for RA, AAS, and PRL^(1,4-8)^.

In conditions associated with LG or salivary gland (SG) dysfunction, including Sjögren’s syndrome (SS), observing the exocrine glands in magnetic resonance imaging (MRI) revealed correlations with volumetric reduction, lower fluid secretion, and other changes^(9)^.

Our objective was to describe three cases of bilateral LG atrophy. The sicca manifestations led to an SS hypothesis; however, the only SS clinically relevant fact identified was the prior chronic use of isotretinoin (an isoform of RA), treatment with AAS in a recreational athlete, and a prolactinoma treated with a dopamine agonist, respectively. The SS investigation was negative in all the three cases, according to the American-European criteria^(10)^.

## CASES REPORT

### Case 1

**Figure 1:**
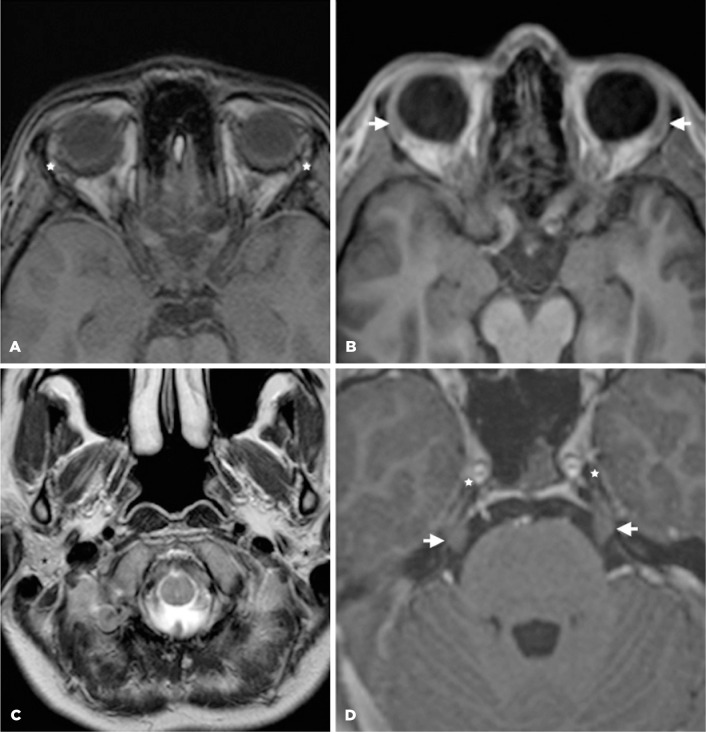
Case 1. A. Axial T1-weigthed magnetic resonance (MR) image, of the upper level of orbits shows the absence of the lacrimal glands (asterisk). B. Axial T1-weighted image, at the same level, in a normal subject (for comparison) shows the usual pattern of the lacrimal glands (white arrows). C. Axial T2-weigthed image shows a normal appearance of the parotid glands of this sequence, with high signal (asterisk). The asymmetry between the right and left sides is due to a slight rotation in the transverse plane. D. T1 axial oblique plane shows the cisternal portion of the trigeminal nerve (arrows) and Meckel’s cave (asterisks).

A 24-year-old white woman presented with DES over the last three years without dry mouth. She reported no comorbidities and no use of medications, except for treatment of acne with RA at 14 and 20 years of age, lasting for six months on both occasions. The ophthalmological examination demonstrated a visual acuity of 1.0 in both eyes (OU); a tear film break-up time (TFBUT) of 2 s in the right eye (OD) and 1 sin the left eye (OS); a grade 5 corneal fluorescein staining in OD and grade 3 in OS, with filamentary keratitis; and a Schirmer test (ST) showed absent tear flow (zero mm) in OU. Moderate MG dysfunction (MGD) with less than 30% of gland drop out, light expressibility, and cloudy oil secretion were observed. The ocular surface disease index (OSDI) was 70.45%, and the whole saliva flow was 0.13 ml/min (normal value, >0.1 ml/min). Serological tests for autoimmune and viral systemic diseases, including anti- Ro/SSA, anti-La/SSB, anti-dsDNA, anti-SM, anti-RNP, antinuclear antibody (ANA), and rheumatoid factor, were negative. A biopsy of her minor lip SG revealed a focus score of zero. MRI revealed the absence of the LG bilaterally (Figure 1A). The average normal LG volume is 0,580 cm^3^.

### Case 2

**Figure 2:**
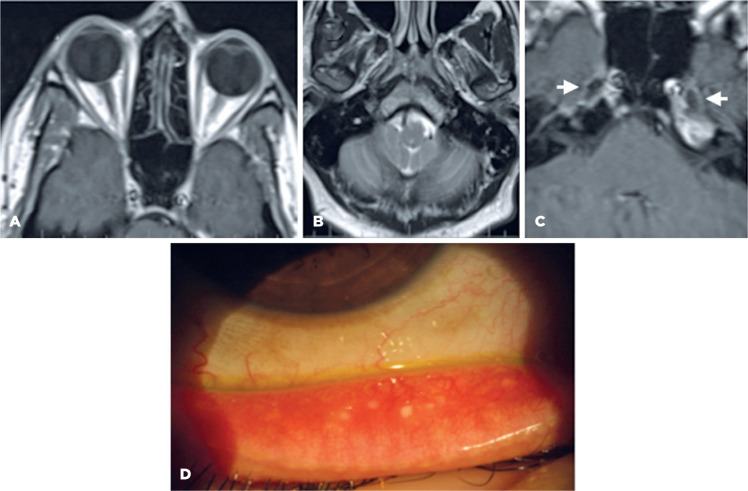
Case 2. A. Axial T1-weigthed magnetic resonance (MR) image at the upper level of the orbits shows the absence of the lacrimal glands. B. Axial T2-weigthed image shows the absence of the parotid gland. C. T1 axial oblique plane shows the cisternal portion of the trigeminal nerve (arrows). D. The tarsal conjunctiva shows hyperemia and conjunctival concretions.

A 32-year-old white man presented with DES and dry mouth for 18 months. Prior to the visit, he received hydroxychloroquine sulfate, corticosteroids, topical cyclosporine, eyedrops, and punctual occlusion for presumed DES secondary to SS, without improvement. His only remarkable previous history was the use of AAS for bodybuilding, as follows: durateston (a solution of four molecules of synthetic testosterone, composed of propionate, fempropionate, isocaproate, and decanoate of testosterone at 30, 60, 60, and 100 mg of each compound per ml, respectively) at one intramuscular injection per week; and stanzonolol (100 mg) via intramuscular injection twice a week. Both were used, as mentioned above, for eight consecutive weeks, two months before the onset of symptoms. No other medications or diseases were reported. The ophthalmological examination demonstrated a visual acuity of 1.0 OU; a TFBUT of 8 s OU; no corneal fluorescein staining; and an ST of 40 mm OU. Examinations of MG and lid margins were normal, but the tarsal conjunctiva exhibited hyperemic and conjunctiva concretions (Figure 2). The OSDI was 90%, and the whole saliva flow was 0.20 ml/min. Serological tests for autoimmune and viral systemic diseases, including anti-Ro/SSA, anti-La/SSB, anti-dsDNA, anti-SM, anti- RNP, ANA, and rheumatoid factor, in addition to blood hormonal assays, were normal. A biopsy of his minor lip SG revealed a focus score of zero. The MRI evidenced that both LGs and the parotid SGs were absent (Figure 2A and B).

### Case 3

**Figure 3:**
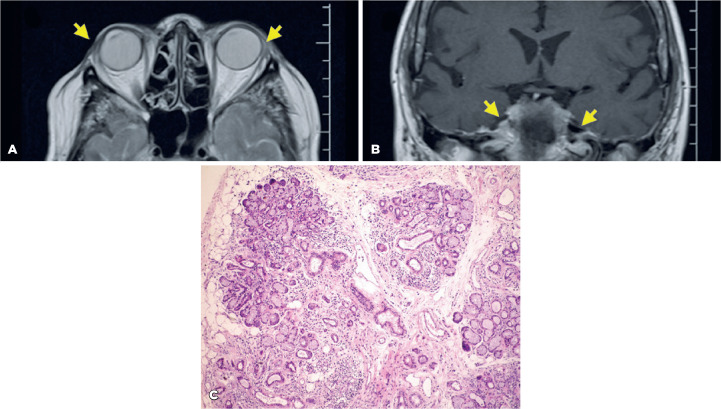
Case 3. A. Axial T1-weigthed magnetic resonance (MR) image at the upper level of the orbits shows the absence of the lacrimal glands (arrows) and the absence of the parotid glands. B. T1 Axial oblique plane shows the cisternal portion of the normal trigeminal nerve (arrows). C. Labial salivary gland biopsy, stained with hematoxylin and eosin, shows acinar hypotrophy, lymphocytic diffuse infiltration, and ductal enlargement.

A 77-year-old female presented with DES for five years, which had worsened 12 months before the visit and was attributed to emotional problems. She was using artificial tears and lacrimal punctal plug occlusion. She mentioned a diagnosis of prolactinoma 30 years prior to this visit, which manifested initially with galactorrhea, further confirmed by laboratory and imaging exams. She had been using carbegoline since that diagnosis. Thyroidectomy and systemic arterial hypertension were treated with Puran T4 and hydroclortiazide, respectively. Her physical exam was not remarkable. Her ocular exam was positive for mild bilateral blepharospasm and mild punctate keratitis. The TFBUT was 30 s and the ST was 5 mm OU. Mild MGD with 20% of gland drop out, light expressibility, and cloudy oil secretion were observed. No changes in the eyelid margin, mucocutaneous junction, or gland orifices were observed. The whole salivary flow was 0.02 ml/min. The laboratory exams were normal, including the prolactin and thyroid stimulating hormone (TSH) levels. The anti-Ro/SSA, anti-La/SSB levels were negative. A biopsy of the lip SG revealed moderate acinar atrophy and mild diffuse lymphocytic infiltration, but no focus score. The MRI analysis revealed bilateral atrophy of the LG and the parotid gland (Figure 3). Moreover, a biopsy of the labial SG showed tissue hypotrophy and diffuse lymphocytic infiltration, but not the typical signs of SS, which are foci of lymphocytic infiltration (Figure 3C).

## DISCUSSION

The observations revealed DES is associated with exposure to RA, AAS, and PRL or, in case 3, with PRL chronic inhibition. RA is used to treat acne vulgaris and as an anti-aging cosmetic, of which DES is a reversible side effect^(7)^. The atrophic LG outcome reported may represent an underdiagnosed event in persistent DES cases. Moreover, the potential association with MGD or other OS changes and discomfort caused by evaporative DES should be considered.

The use of AAS, which causes side effects as DES, can be more difficult to correlate in this setting because many patients omit this information. Many side effects are being reported, some severe, but this drug’s popularity and its abuse are rampant among teenagers and adults^(8)^. The causes of its side effects are associated with disturbance of the hypothalamus-hypophysis axis, the impact on the brain’s neuropeptides, and calcium imbalance; moreover, its effects on several organs have been described, including the liver, pancreas, and testis, but the association with LG atrophy was not reported previously, to the best of our knowledge^(11)^.

The association between PRL and DES and SS is attributed to its bimodal trophic effect on the exocrine glands and the proinflammatory actions of this hormone^(1,4,6)^. In the case reported here, the long period of existence of a prolactinoma, treatment with a pharmacological inhibitor, and lowering the sex hormones could have induced inflammation in the LG and SG at the very beginning of the PRL rise. Further, it treatment may have caused atrophy and PRL inhibition over the subsequent decades, and a combined negative effect of sex hormone senescence and PRL inhibition in older age. The exact natural history of this case is unclear.

In summary, prospective cohort studies are required to support the atrophic collateral effects of excessive exposure to RA, AAS, and PRL on the LG, mimicking SS. Based on the frequency of those conditions, they must be included in differential diagnoses of DES and SS.
